# Supplementary material for: Biphasic activation of β-arrestin 1 upon interaction with a GPCR revealed by methyl-TROSY NMR
Source: Nat Commun. 2021 Dec 9;12:7158. doi: 10.1038/s41467-021-27482-3 (PMC8660791; doi:10.1038/s41467-021-27482-3)
Supplement: Supplementary file 1 — Supplementary Information [file 41467_2021_27482_MOESM1_ESM.pdf]

Supplementary Information

Biphasic activation of β-arrestin 1 upon interaction with a GPCR revealed by methyl-TROSY NMR

Shiraishi *et al.*

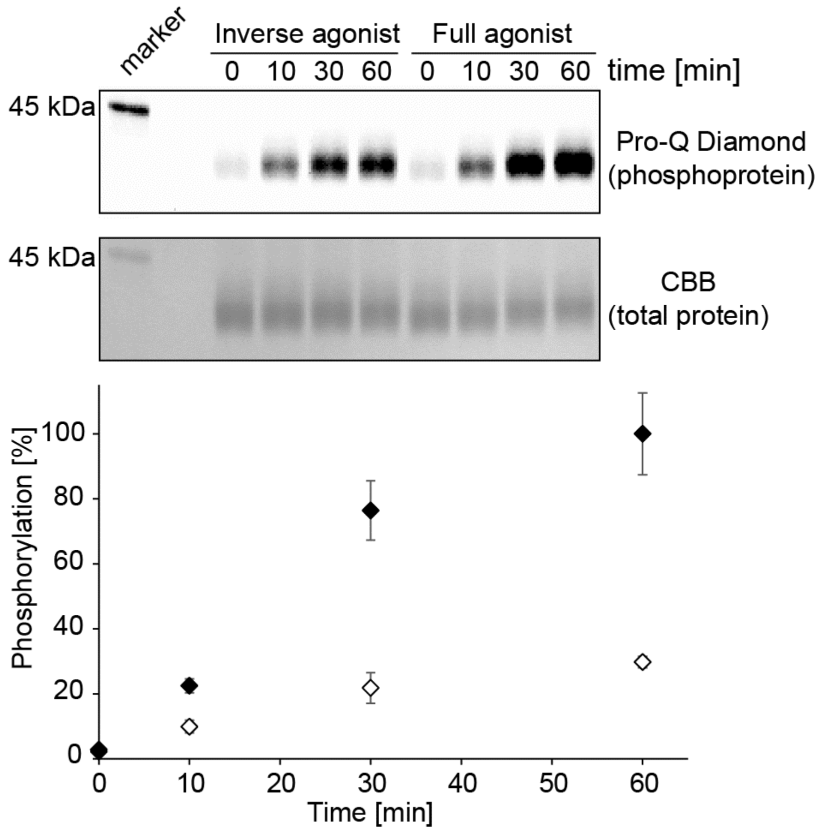

Supplementary Figure 1 | GRK2-mediated phosphorylation of prepared β_2V_2R in rHDLs.

Reaction mixtures at the indicated time points were analyzed by SDS-PAGE with Pro-Q Diamond and Coomassie Brilliant Blue staining. Data represent mean values ± standard deviation of three independent experiments.

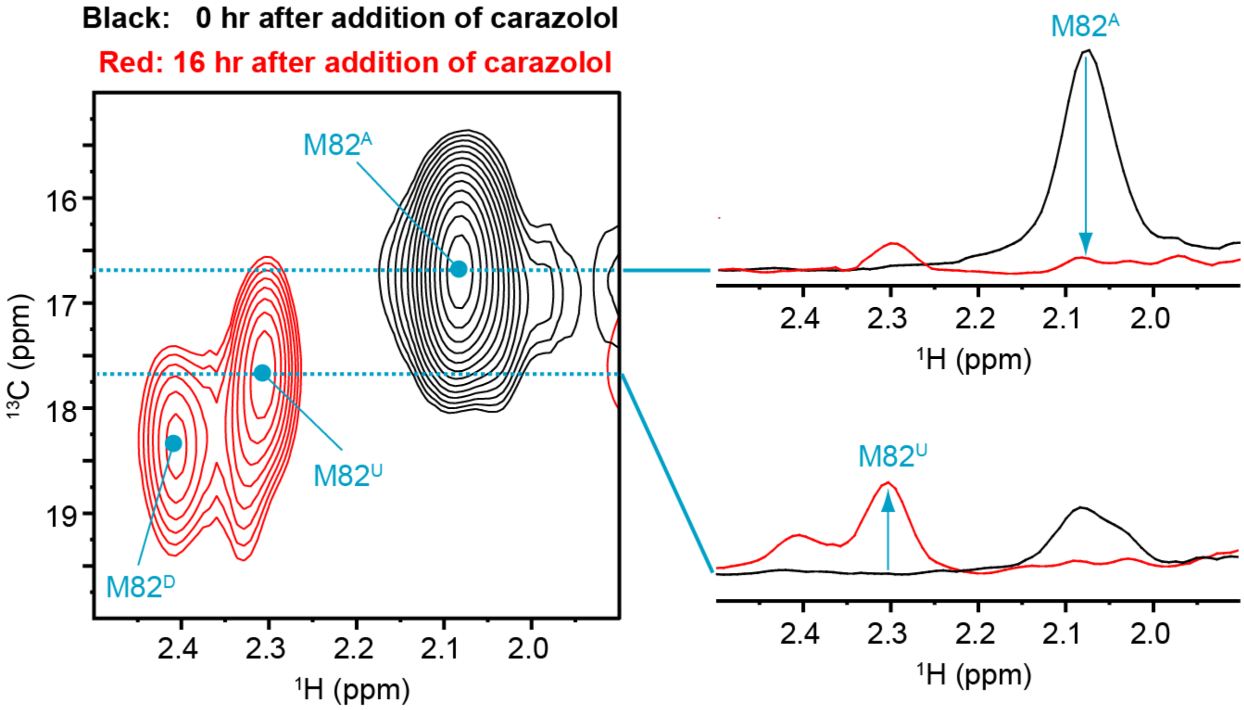

Supplementary Figure 2 | Confirmation of ligand exchange as monitored by the resonances from the M82 methyl group of phosphorylated β_2V_2R in rHDLs. Overlay of ^{1}H-^{13}C HMQC spectra of [^{2}H-9AA, αβγ-^{2}H, ε-^{13}C-Met] phosphorylated β_2V_2R bound to formoterol immediately (black) and 16 h (red) after carazolol addition. The acquisition time was 3 h for each spectrum. Previous studies reported that the M82 methyl group resonances exhibited distinctly different chemical shifts between the formoterol-bound ($M82^{A}$) and carazolol-bound ($M82^{U}$ and $M82^{D}$) states [1, 2]. Cross sections of the resonances corresponding to $M82^{A}$ and $M82^{U}$ are shown on the right.

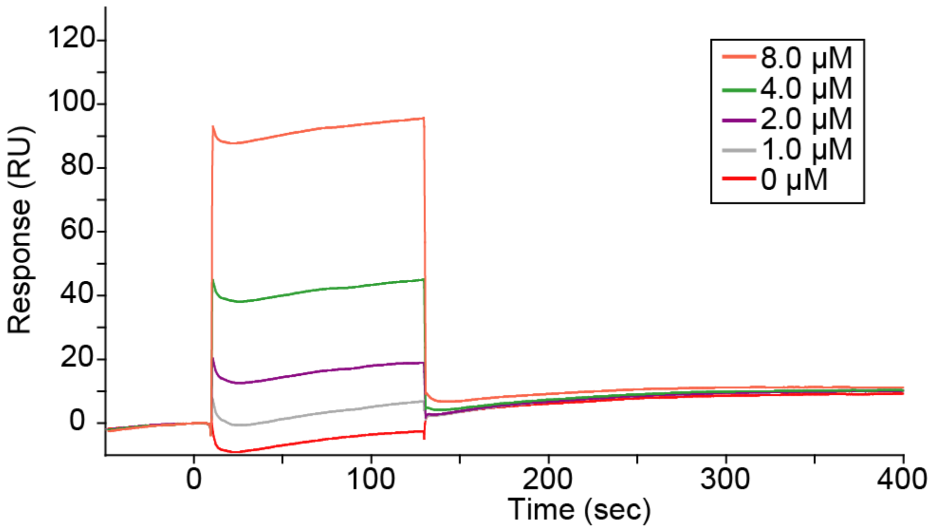

Supplementary Figure 3 | Effects of non-specific binding of βarr1 to the sensor chip surface. Overlay plots of sensorgrams obtained upon injections of 0, 1.0, 2.0, 4.0, and 8.0 μM βarr1 into flow cells without immobilized molecules.

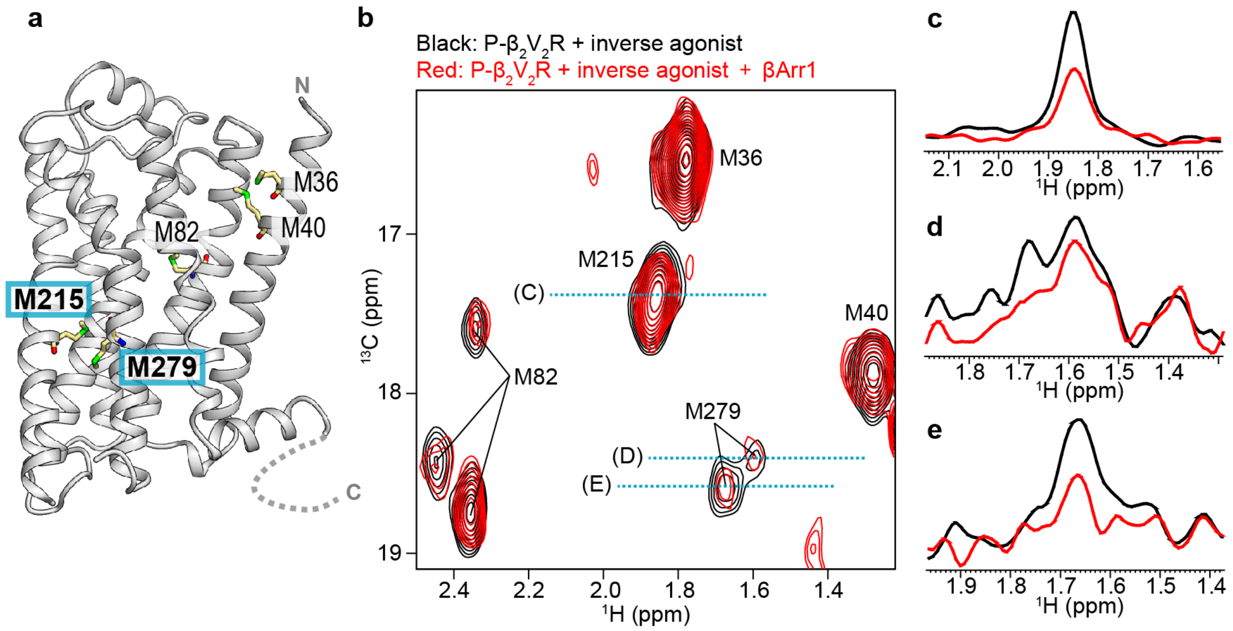

Supplementary Figure 4 | Effect of βarr1 binding on the conformation of the transmembrane region of phosphorylated β_2V_2R bound to an inverse agonist. (a) Distribution of the NMR probes on the structure of the β_2AR TM region. The crystal structure of β_2AR bound to an inverse agonist, carazolol, (PDB ID: 2RH1) [3], is shown as a ribbon model, and the methionine residues observed in the experiments are depicted by sticks. M215 and M279, with resonances that reportedly exhibit significant chemical shift changes upon βarr1 binding in the full agonist-bound state, are highlighted [4]. (b) Overlay of ^{1}H-^{13}C HMQC spectra of [^{2}H-9AA, αβγ-^{2}H, ε-^{13}C-Met] phosphorylated β_2V_2R bound to the inverse agonist (black) and in the complex with βarr1 (red). (c–e) ^{1}H-1D cross-sections of resonances from M215 and M279. The intensity reductions are probably due to the increased apparent molecular weight upon βarr1 binding.

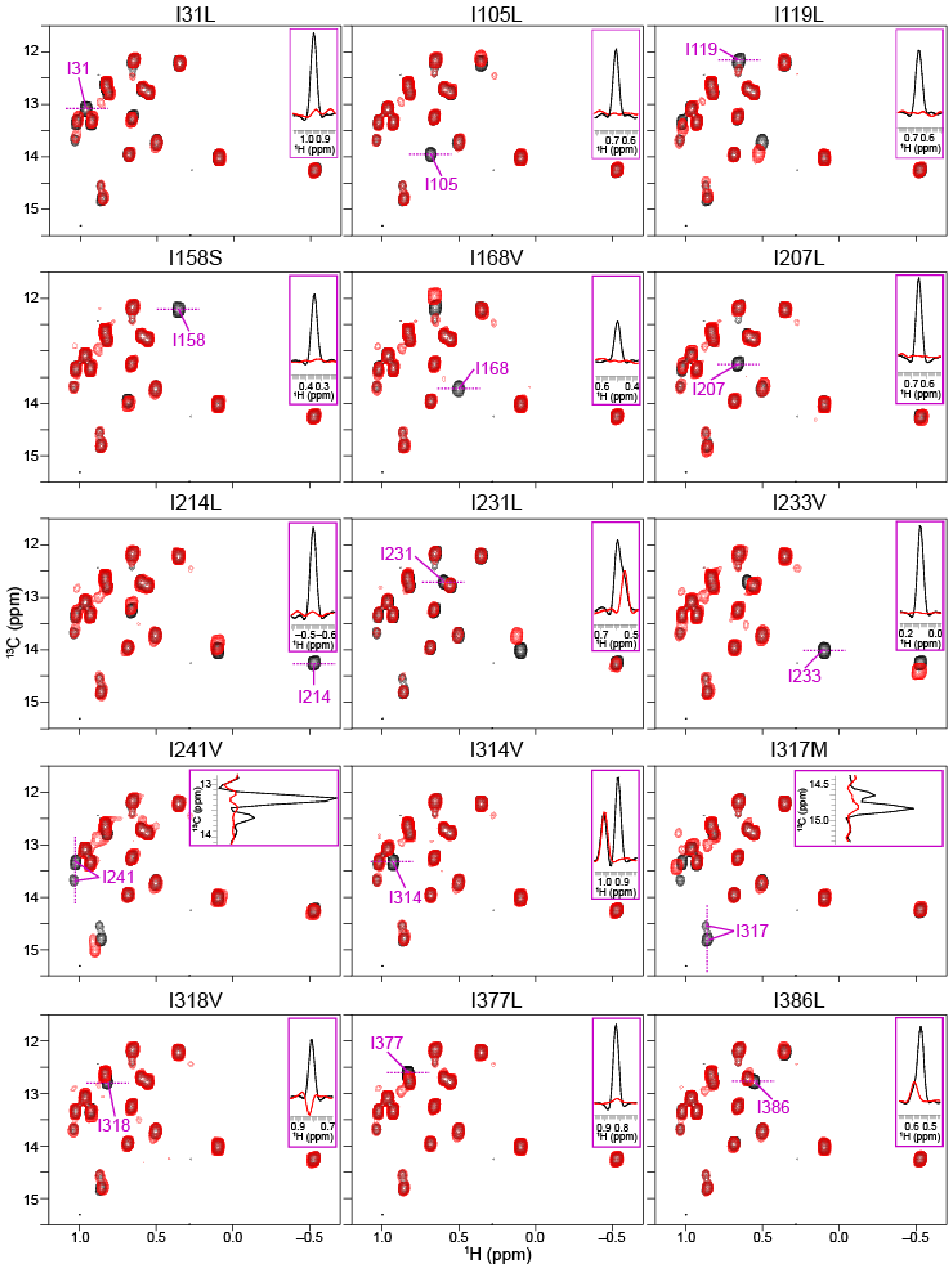

Supplementary Figure 5 | Assignment of Ileδ1 methyl resonances of [u-^{2}H, Ileδ1-^{13}C^{1}H$_3$] βarr1. ^{1}H-^{13}C HMQC spectra of βarr1 and its mutants, in which isoleucine residues are

substituted with other amino acids, are overlaid in black and red, respectively. Resonances assigned to mutated residues are labeled. Cross sections of assigned resonances at the dashed lines are shown in the insets.

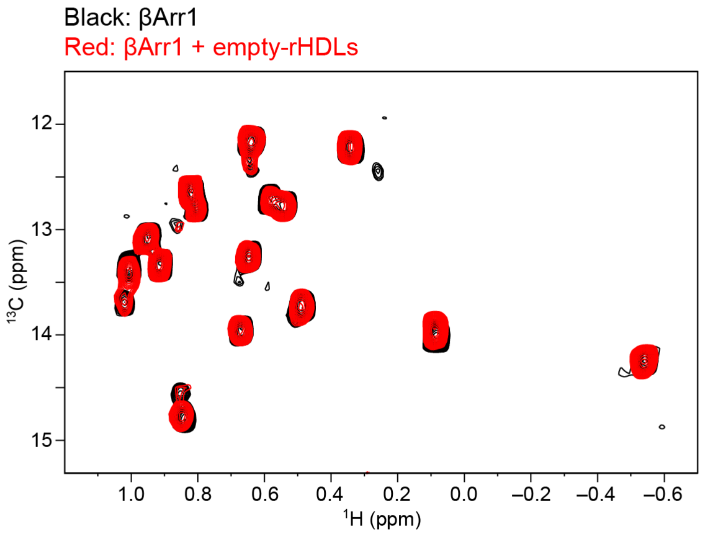

Supplementary Figure 6 | Effect of the addition of rHDLs without receptors on the βarr1 conformation. Overlay of ^{1}H-^{13}C HMQC spectra of [u-^{2}H, Ileδ1-$^{13}C^{1}H_{3}$] βarr1 in the basal state (black) and in the presence of an excess amount of empty rHDLs (red).

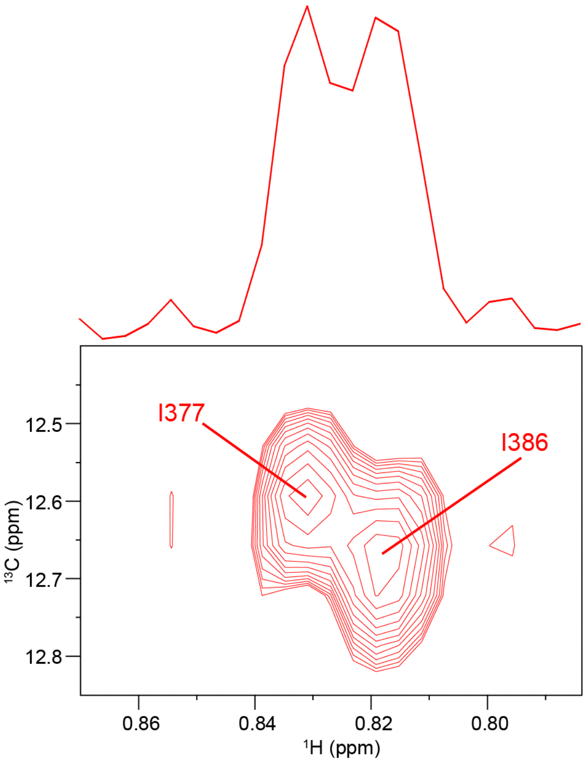

Supplementary Figure 7 | Magnified view of the resonances from I377 and I386 of βarr1 in the complex with phosphorylated β_2V_2R in rHDLs bound to the full agonist. No apodization function was multiplied to the ^{1}H dimension prior to the Fourier transformation. The projection is displayed above the spectrum.

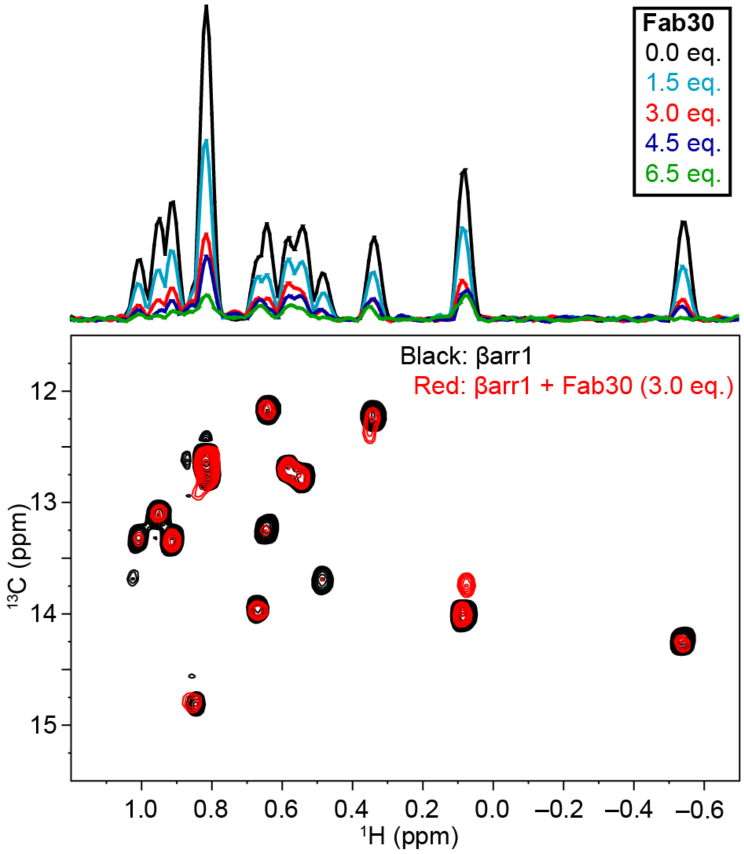

Supplementary Figure 8 | βarr1–Fab30 interactions in the absence of β$_2$V$_2$R. Overlay of ^{1}H-^{13}C HMQC spectra of [u-^{2}H, Ileδ1-^{13}C^{1}H$_3$] βarr1 in the basal state (black) and in the presence of 3.0 molar equivalents of Fab30 (red). Overlaid projections in the presence of 0.0 (black), 1.5 (cyan), 3.0 (red), 4.5 (violet), and 6.5 (green) molar equivalents of Fab30 are displayed above the spectra.

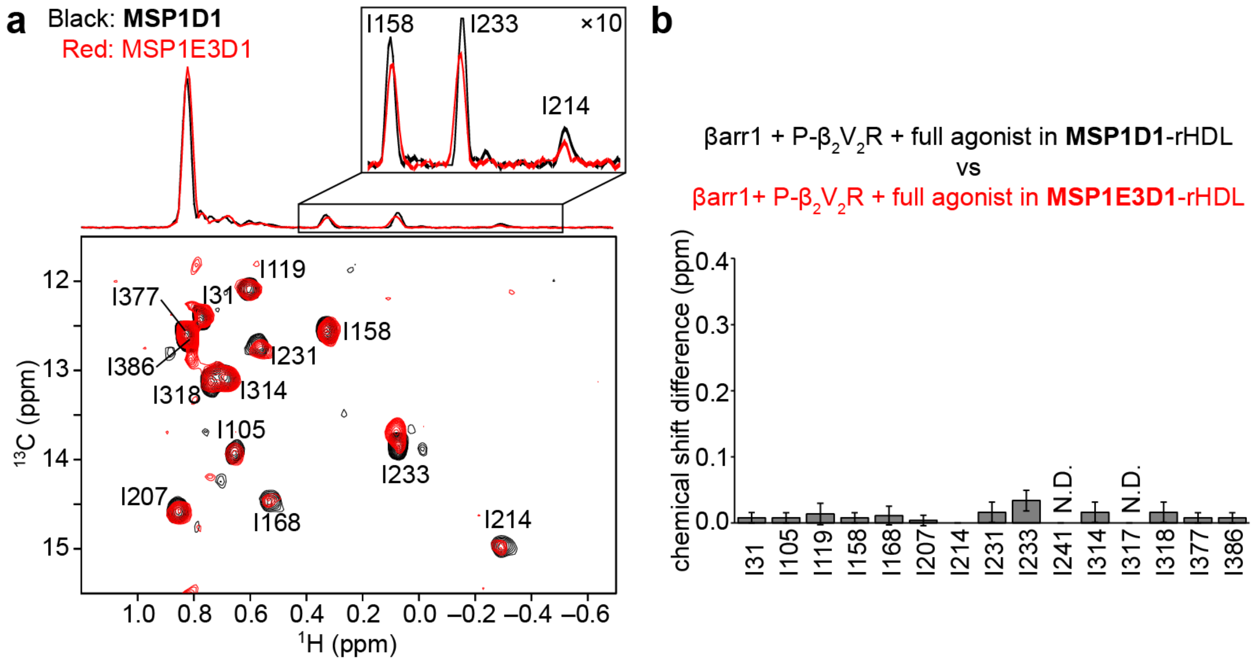

Supplementary Figure 9 | Effects of the rHDL sizes on the βarr1 conformation in the complex with phosphorylated β_2V_2R in rHDLs bound to the full agonist. (a) Overlay of ^1H-^{13}C HMQC spectra of [u-^2H, Ileδ1-$^{13}C^1H_3$] βarr1 in the complex with phosphorylated β_2V_2R bound to the full agonist, in rHDLs assembled with MSP1D1 (black) and rHDLs assembled with MAP1E3D1 (red). The ^1H 1D projections are shown above the spectra, and a magnified view of the projection including resonances from I158, I214, and I233 is shown in the inset. (b) Normalized chemical shift differences of βarr1 methyl groups between the complex with phosphorylated β_2V_2R bound to the full agonist in rHDLs assembled with MSP1D1 and the complex with phosphorylated β_2V_2R bound to the full agonist in rHDLs assembled with MSP1E3D1. The error bars were calculated based on the digital resolution of the spectra, as described in Methods section.

Supplementary Table 1 | Oligonucleotide sequences used to generate βarr1 variants

	DNA sequence (5'-3')
I31L_forward	CTTTGTGGACCACCTCGACCTCGTGGACCCT
I31L_reverse	AGGGTCCACGAGGTCGAGGTGGTCCACAAAG
I105L_forward	GCAGGAACGCCTCCTCAAGAAGCTGGGCG
I105L_reverse	CGCCCAGCTTCTTGAGGAGGCGTTCCTGC
I119L_forward	CCCTTTCACCTTTGAGCTCCCTCCAAACCTTCC
I119L_reverse	GGAAGGTTTGGAGGGAGCTCAAAGGTGAAAGGG
I158S_forward	GAATTTGGAGGAGAAGAGCCACAAGCGGAATTCTG
I158S_reverse	CAGAATTCCGCTTGTGGCTCTTCTCCTCCAAATTC
I168V_forward	CTGTGCGTCTGGTCCTCCGGAAGGTTCAGTATG
I168V_reverse	CATACTGAACCTTCCGGAGGACCAGACGCACAG
I207L_forward	CCTCTCTGGATAAGGAGCTCTATTACCATGGAG
I207L_reverse	CTCCATGGTAATAGAGCTCCTTATCCAGAGAGG
I214L_forward	CCATGGAGAACCCCTCAGCGTCAACGTCC
I214L_reverse	GGACGTTGACGCTGAGGGGTTCTCCATGG
I231L_forward	GGAGACGGTGAAGAAGCTCAAGATCTCAGTGC
I231L_reverse	GCACTGAGATCTTGAGCTTCTTCACCGTCTCC
I233V_forward	GGTGAAGAAGATCAAGGTCTCAGTGCGCCAG
I233V_reverse	CTGGCGCACTGAGACCTTGATCTTCTTCACC
I241V_forward	GCGCCAGTATGCAGACGTCGTCCTTTTCAACAC
I241V_reverse	GTGTTGAAAAGGACGACGTCTGCATACTGGCGC
I314V_forward	GCCAACCGTGAGGTCCTGGGGATCATTG
I314V_reverse	CAATGATCCCCAGGACCTCACGGTTGGC
I317M_forward	CCGTGAGATCCTGGGGATGATTGTTTCCTAC
I317M_reverse	GTAGGAAACAATCATCCCCAGGATCTCACGG
I318V_forward	GAGATCCTGGGGATCGTTGTTTCCTACAAAGTG
I318V_reverse	CACTTTGTAGGAAACAACGATCCCCAGGATCTC
I377L_forward	CCAGTAGATACCAATCTCCTAGAACTTGACACAAATGATG
I377L_reverse	CATCATTTGTGTCAAGTTCTAGGAGATTGGTATCTACTGG
I386L_forward	GACACAAATGATGACGACCTTGTATTTGAGGACTTTGC
I386L_reverse	GCAAAGTCCTCAAATACAAGGTCGTCATCATTTGTGTC

Supplementary Note 1

Effects of the rHDL sizes on the βarr1 conformation in the complex with phosphorylated β_2V_2R in rHDLs bound to the full agonist.

To determine whether differences in the rHDL sizes could affect the βarr1 conformation, the NMR spectrum of βarr1 in the complex with phosphorylated β_2V_2R bound to the full agonist in rHDLs assembled with MSP1D1 was compared with that in rHDLs assembled with MSP1E3D1 (Supplementary Fig. 9). The observed chemical shift differences were small (<0.05 ppm), suggesting that the βarr1 conformation in the complex with phosphorylated β_2V_2R bound to the full agonist in rHDLs assembled with MSP1D1 was almost identical to that in rHDLs assembled with MSP1E3D1 (Supplementary Fig. 9b). More intense resonances were obtained when using MSP1D1, probably because the rHDLs assembled with MSP1D1 are smaller than those with MSP1E3D1 (Supplementary Fig. 9a). Therefore, except for the NMR experiment in Supplementary Fig. 9, MSP1D1 was used to construct rHDLs.

Supplementary References

1. Kofuku, Y., et al., *Efficacy of the β_2-adrenergic receptor is determined by conformational equilibrium in the transmembrane region.* Nat. Commun., 2012. **3**: p. 1045.
2. Kofuku, Y., et al., *Functional dynamics of deuterated β_2-adrenergic receptor in lipid bilayers revealed by NMR spectroscopy.* Angew. Chem. Int. Ed. Engl., 2014. **53**(49): p. 13376-13379.
3. Rosenbaum, D.M., et al., *GPCR engineering yields high-resolution structural insights into β_2-adrenergic receptor function.* Science, 2007. **318**(5854): p. 1266-73.
4. Shiraishi, Y., et al., *Phosphorylation-induced conformation of β2-adrenoceptor related to arrestin recruitment revealed by NMR.* Nat. Commun., 2018. **9**(1): p. 194.
